# Realising the potential human development returns to investing in early and maternal nutrition: The importance of identifying and addressing constraints over the life course

**DOI:** 10.1371/journal.pgph.0000021

**Published:** 2021-10-13

**Authors:** Chris Desmond, Agnes Erzse, Kathryn Watt, Kate Ward, Marie-Louise Newell, Karen Hofman, on behalf of the INPreP group

**Affiliations:** 1 Faculty of Health Sciences, SAMRC/Wits Centre for Health Economics and Decision Science, PRICELESS, University of Witwatersrand School of Public Health, Johannesburg, South Africa; 2 Centre for Rural Health, University of KwaZulu-Natal, Durban, KwaZulu-Natal, South Africa; 3 Faculty of Medicine, School of Human Development and Health, University of Southampton, Southampton, United Kingdom; Penn State Health Milton S Hershey Medical Center, UNITED STATES

## Abstract

The benefits of interventions which improve early nutrition are well recognised. These benefits, however, only accrue to the extent that later life circumstances allow. Consequently, in adverse contexts many of the benefits will never be realised, particularly for the most vulnerable, exacerbating inequality. Returns to investment in early nutrition could be improved if we identified contextual factors constraining their realisation and interventions to weaken these. We estimate cost and impact of scaling 10 nutrition interventions for a cohort of South African children born in 2021. We estimate associated declines in malnutrition and mortality, and improvements in years of schooling and future earnings. To examine the role of context over the life-course we estimate benefits with and without additional improvements in school quality and employment opportunities by socio-economic quintile. Scale up reduces national stunting (height for age < = -2SD) rates among children at 24 months by 3.18 percentage points, implying an increase in mean height for age z-score (HAZ) of 0.10, and 53,000 years of additional schooling. Quintile 1 (the poorest) displays the largest decline in stunting, and largest increase in mean HAZ. Estimated total cost of increasing coverage of the interventions for the cohort is US$90 million. The present value of the additional years of schooling is estimated at close to US$2 billion. Cost-benefit ratios suggest the highest return occurs in quintile 5 (1:23). Reducing inequality in school quality closes the gap between quintile 5 and the lower quintiles. If school quality and labour force participation were equal the highest returns are in quintile 1(1:31). An enabling environment is key to maximising human development returns from investing in early nutrition, and to avoid exacerbating existing inequality. Therefore, particularly for children in adverse conditions, it is essential to identify and implement complementary interventions over the life course.

## Introduction

South Africa faces a high burden of child undernutrition, 2016 data indicate that 27% of children under 5 years of age were stunted (height for age z-score (HAZ)<-2SD), children in the poorest wealth quintile had the highest proportion of stunting, 36.3% compared to 12.5% in the richest quintile [1]. Interventions which improve early nutrition have the potential to improve outcomes over the life-course [2,3], thereby generating high returns [4], but these returns are not automatic and only accrue to the extent that later life circumstances allow. Therefore, to realise the potential value of investments in early nutrition, we must identify and address the constraints on development over the life course. We outline how a failure to consider later life constraints can lead to the systematic undervaluing of early nutrition interventions for the most vulnerable who have to contend with multiple constraints in childhood and as adults. And further, how failure to address these constraints can lead to nutrition interventions exacerbating existing inequality. Early nutrition here refers to adequate and appropriate maternal nutrition preconception and during pregnancy, and appropriate child nutrition in the first two years of life.

We apply a novel human development framework to highlight the importance of identifying how contexts can be improved to increase returns on investments in early nutrition and reduce inequality in human development outcomes. This approach requires a shift from the piecemeal consideration of interventions in narrowly defined sectors and life stages, to the coordinated cross-sectoral planning of investments across the life course.

We illustrate our approach with a case study of possible returns on investment in early nutrition interventions in South Africa and how these vary across socio-economic groups. We focus on the interactions between early nutrition, which protects human development potential, school quality which influences the realisation of protected potential, and labour market opportunities which shape the utilisation of realised potential. As one of the most unequal countries in the world, with a gini coefficient of 0.63 in 2015, South Africa provides a powerful case study of how differences in early nutrition interact with differences in living conditions across childhood and into adulthood to shape human development outcomes [5].

While our focus is on nutrition and a country case study, the argument has implications for a range of early-life interventions and cross-country comparisons of nutrition and Early Childhood Development (ECD) interventions. Without an explicit focus on constraints over the life course, we run the risk of mistakenly confusing the currently realisable returns of nutrition and ECD interventions with the potential value of these interventions, leading to the troubling conclusion that investing in the early development of children in adversity is of lower value than investing in those living in more supportive environments. Perhaps more troubling is that if constraints hindering the realisation of potential are not identified and addressed, those most in need will benefit less per dollar invested than their better off peers.

### Determinants and consequences of early nutrition: The role of context

**Fig 1:**
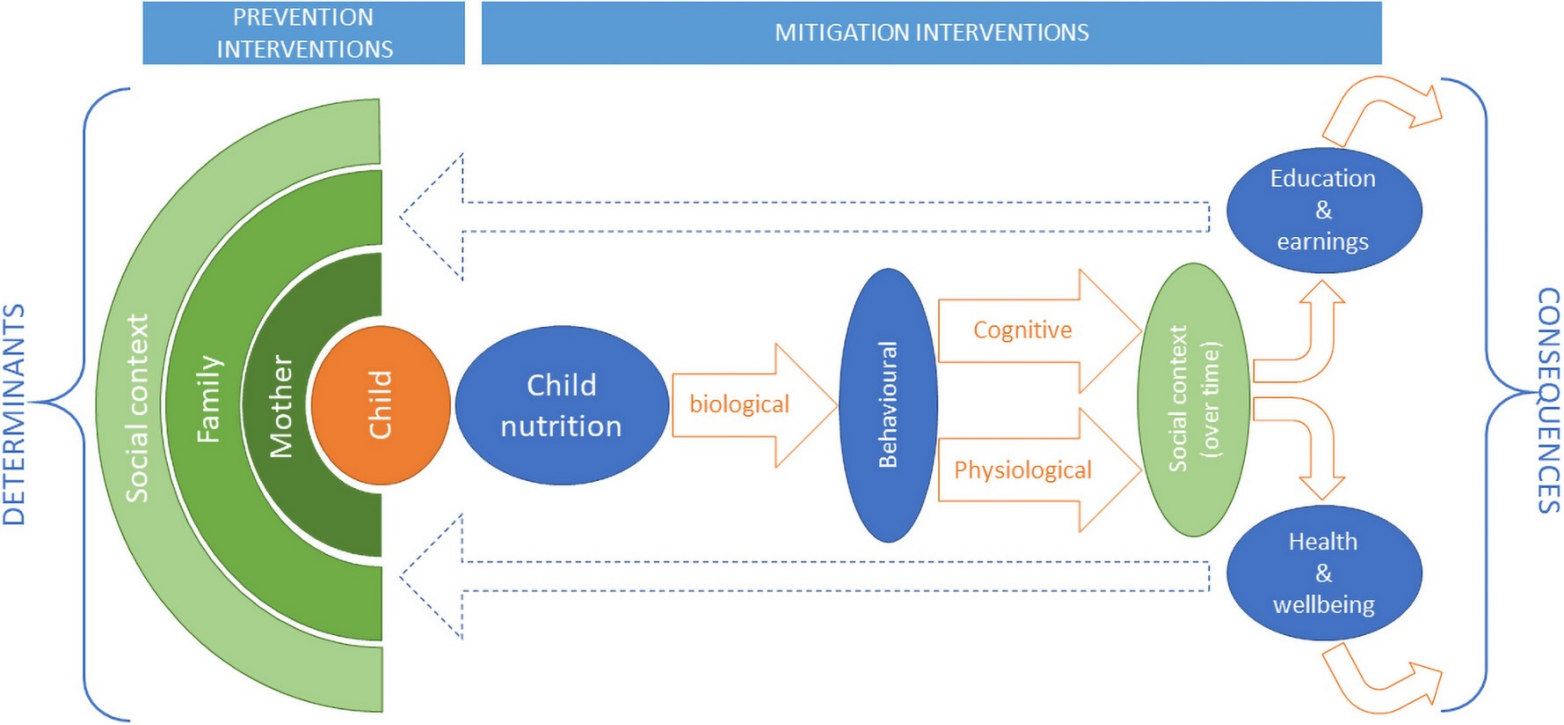
The determinants and consequences of nutritional status in the first 1000 days plus. The immediate and short-term biological consequences of unmitigated poor early nutrition include impacts on cognitive development and physiological health, including mortality, with some variation associated with child level differences, such as genetics [6,8,9]. The long-term implications of these consequences are largely dependent on whether, and how quickly, children access appropriate interventions [10,11]. Without intervention, the cognitive consequences of poor nutritional status can make school more difficult, often leading to early drop-out, and consequent lower earnings [12]. Positive caregiver responses such as accessing appropriate services, and providing adequate child stimulation can compensate for nutritional deficits, thus limiting negative biological consequences of poor early nutrition [13,14]. Conversely, caregivers attempting to protect a child they perceive as weak because of their nutritional status may inadvertently worsen biological outcomes through under-stimulation [15–17]. Early interventions have the greatest potential to reverse the consequences of early malnutrition, but many later-life interventions, such as quality schooling, can mitigate the effects of early malnutrition on health, education and associated earnings, and wellbeing [18–20]. The later life outcomes of adults who experienced poor early nutrition are shaped by their socio-economic context, such as the labour market. These later life outcomes shape the family circumstances of the next generation of children. This leads to intergenerational consequences of poor early nutrition [8,21].

Undernutrition, overnutrition and micronutrient deficiencies in the first 1000 days each have the potential to influence a range of immediate and long-term development outcomes, including growth and cognitive development [6]. Young children’s nutritional status is shaped by interacting factors including genetics, pre- and post-conception maternal health and wellbeing, household socio-economic status, community norms, and national policy [2,7]. The severity of the consequences of poor early nutritional status are determined by a child’s current and future socio-economic context, including, the quality and quantity of nutrition later in life, caregivers’ response to children’s nutritional status, and the nature of childhood services they can access. Fig 1 provides a summary of the determinants and consequences of early nutritional status.

It is well recognised that to capture the value of interventions to improve early nutrition, the evaluation space must be broadly defined to account for multiple possible outcomes across the life course [12]; limiting economic valuation to health outcomes, risks undervaluing such interventions [4]. What is not often highlighted is that the value of early nutrition interventions is determined by the current and future context in which their beneficiaries live. Those who already live in contexts which allow the full benefits of early nutrition to be realised will experience the largest lifetime benefits from a given improvement in early nutrition increasing inequality. For example, productivity returns are a function of baseline earning so larger increases in earning will accrue to those with the highest baseline earning in the absence of intervention [4,11]. Without complementary interventions, we should anticipate lower returns to investments in early nutrition for women, disabled people, rural populations and people in poorer countries.

### A human development framework

**Fig 2:**
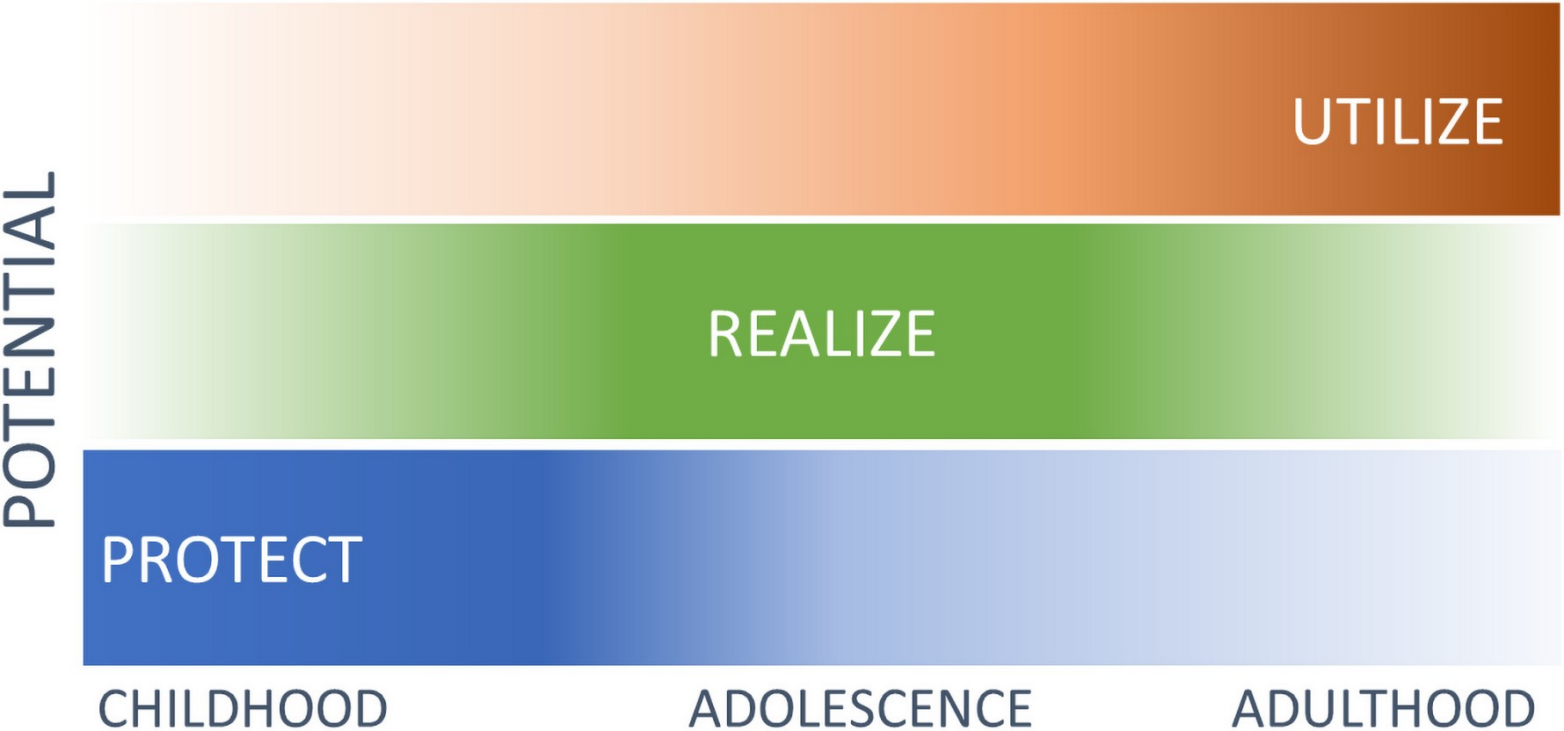
Human development framework. The framework focuses on human development, rather than intermediary outcomes. It includes three interacting types of influence on human development: The protection of potential; the realisation of potential; and the utilisation of potential. To be realized, potential must be protected, and to be utilized, potential must be realized. Influences which protect potential are concentrated in early life, those which lead to the realisation of potential cluster in mid-childhood and adolescence; and those which determine the possibility of utilising potential are concentrated in adulthood. While concentrated in particular periods, influences of each type can be found at every life stage. This framework is an adaptation of Nussbaum’s work on basic, internal, and combined capabilities [22]. Nussbaum focused on the interaction of individual development and the contexts in which people live, this framework deviates from Nussbaum in its treatment of biological development which she assumed was essentially automatic [22].

Our novel human development framework (Fig 2) highlights the interactions of interventions over the life course which protect potential, facilitate its realisation, and allow or promote its utilisation. The framework outlines why the benefits of interventions to protect potential early in life will be large in contexts that give children the opportunity to realise and utilise that potential, but small in contexts where these opportunities are absent or constrained. Complementary interventions which address constraints on the realisation of potential, such as improved schooling and protection from violence, will improve returns. Similarly, those which allow its utilisation, such as policies that eliminate racial and gender discrimination in the workforce will also improve returns.

The framework helps explain why interventions early in life often fade over time for children living in adversity [12], and do not always yield the high returns predicted by the Heckman Curve [23]. It suggests that if future investments are low or absent, the mechanisms of self-productivity and dynamic complementarity which drive Heckman’s result [24], cannot operate, and the long-term benefits of improving developmental outcomes in early life will be low.

To illustrate the implications of this framework we estimate the returns to investing in interventions to improve early nutrition in South Africa. Even 25 years post-apartheid, severe inequality remains in early nutrition, educational opportunities and almost every human development outcome [25–27]. In this context, an evaluation of interventions to improve early nutrition must consider barriers to development over the life course, to avoid recommendations which would entrench inequality. This approach could be applied either to other countries or to cross-country analyses.

## Methods

We estimate the impact in South Africa of scaling to 90% coverage 10 nutrition-specific interventions for women in the reproductive period and their children 0–24 months identified by Bhutta et al (Table 1), the evidence for which has strengthened in recent years [2,28,29]. We model the consequences of scale up for improvements in nutrition, reductions in mortality, and consequential increases in schooling, and associated productivity returns, separately for five socio-economic quintiles of a hypothetical cohort of children born in 2021. Quintile 1 is the poorest and quintile 5 the richest.

**Table 1:** Baseline intervention coverage and socio-economic context in South Africa.

Interventions	Baseline coverage by quintile 2020 (%)				
	Q1	Q2	Q3	Q4	Q5
Folic acid supplementation or fortification (all women 15–49) ^a^	72	77	80	79	82
Calcium supplementation in pregnancy ^a^	18	19	20	20	21
Multiple micronutrient supplementation in pregnancy ^b^ (current coverage of Iron supplementation in pregnancy ^c^)	0 (88)	0 (91)	0 (91)	0 (93)	0 (88)
Balanced energy supplementation ^b^	0	0	0	0	0
Breastfeeding promotion ^b^	6	6	6	6	6
Complementary feeding—supplementary feeding and education ^d^	47	44	45	56	62
Vitamin A supplementation ^e^	70	73	73	71	74
Zinc supplementation ^b^	0	0	0	0	0
Therapeutic feeding for severe wasting (SAM) ^a^	40	43	45	44	46
Treatment for moderate acute malnutrition (MAM) ^a^	22	24	25	25	26
**Socio-economic context**					
School quality (proxy: matric bachelor pass rate %) [30]	37	39	40	45	61
Average years of schooling ^f^	10	10.5	10.9	11.1	11.5
School completion rate ^f^	0.3	0.4	0.47	0.53	0.65
Returns to education (baseline %) ^g^	15.6	15.8	16.0	16.5	18.4
Labour force participation rate ^f^	0.4	0.4	0.4	0.5	0.6

^a^ Baseline coverage levels of selected nutrition interventions without publicly available data were elicited from South African Maternal Nutrition and Child Health experts during an expert panel review meeting conducted as part of this study in July 2016. The authors used these estimates in conjunction with SADHS data on ANC visits to calculate appropriate coverage by quintile [31];

^b^ Currently not provided in the public sector;

^c^ Percentage of women who took iron tablets during the pregnancy of their most recent live birth [1];

^d^ Proxy used: Among all children 6–23 months, percentage fed minimum dietary diversity [1];

^e^ Among all children age 6–59 months: Percentage given vitamin A supplements in past 6 months [1];

^f^ Author calculations based on data [32];

^g^ Author calculations based on data [33,34].

Each of the quintile models is calibrated to reflect the environment faced by the 20% (230,000 children) of the cohort (children born in 2021) in that quintile. Environmental differences modelled include baseline coverage of the nutrition interventions, nutritional status, health outcomes, poverty rates, school quality, and labour market opportunities. Assumptions and data sources are summarised in Table 1, (full details in S1 Appendix).

The impact and cost of improving nutrition intervention coverage in each quintile model was estimated using the Lives Saved Tool (LiST) [35]. LiST reports the decline in stunting (height for age z-score (HAZ)<-2 standard deviations below the median) associated with increased coverage of the package of interventions. As done by Fink et al [36], and Alderman et al [4], we assume that the benefits of improved nutrition are not limited to those children who would have been stunted in the absence of intervention but will accrue to all children whose nutrition is improved. We calculate the shift in a normally distributed growth curve required to produce the decline in stunting reported by LiST. This provides an estimate of the average increase in HAZ. Following Fink et al [36], we convert this increase to an estimate of the increase in years of schooling by assuming a linear relationship between the two: 1 standard deviation improvement in HAZ is assumed to increase completed schooling by 0.48 years, based on the analysis by Adair et al of five birth cohorts in low- and middle-income countries [37]. In sensitivity analysis we consider a 50% larger and 50% smaller impact, and we limit the improvements in schooling only to those children who would have been stunted in the absence of intervention.

To estimate the productivity returns associated with the increase in years of schooling in each quintile, we estimate the present value of life-time earnings under four scenarios: (1) a baseline with no additional intervention; (2) nutrition interventions scaled to 90%; (3) nutrition interventions scaled to 90%, and improved school quality; (4) nutrition, and equitable school quality, and equitable labour market opportunities. Improved school quality is defined as improving the returns to education in each of quintiles 1–4 by 50% of the difference between current returns in that quintile versus quintile 5 (S4 Appendix). Equitable labour markets are defined as individuals in each quintile facing the same probability of employment currently associated with individuals in quintile 5. Productivity returns are estimated based on a gradual (1% per annum) increase in real income until the cohort enter the labour market for 40 years of work. Quintile and scenario estimates are based on appropriate changes to the average years of schooling, returns to education and employment probabilities. The difference between the baseline (scenario 1) and scenarios 2–4 provides an estimate of the present value of the returns to investment. The calculation of productivity returns was made in Excel, details of the assumptions, formula and data sources for each scenario are provided in S1–S5 Appendices.

We report total implementation cost of the nutrition interventions from the perspective of the intervention provider, and include the cost of drugs, materials, staffing, and the use of existing facilities (S2 Appendix). We do not include the cost of new facilities as it is unlikely they would be needed for integrated services provided within a system which already has high coverage. We include the cost of increased years of schooling, calculated separately based on the current level of expenditure on schooling in each quintile.

We did not estimate the costs associated with improving school quality or labour market opportunities, nor did we include the cost of the infrastructure already in place, such as the higher quality schools attended by children in quintile 5. If only the costs of new interventions are included, our analysis will always favour those who already have access to resources, because of positive interactions.

### Ethics statement

This modelling study drew upon several publicly available anonymised datasets. The study did not directly or indirectly recruit or involve human subjects, and therefore did not require approval from an ethics review board.

## Results

Table 2 reports the cost of improving nutrition intervention coverage to 90%, and the cost associated with consequential increases in years of schooling, by quintile and nationally. The costs are highest in quintiles 1 and 2 because baseline coverage rates were on average lower, and the need for targeted interventions higher. The total cost of increasing coverage nationally is estimated to be US$90.0 million per cohort. The cohort cost is slightly higher than the annual cost as some interventions (zinc supplementation for example) are received for more than 1 year. The cost of increased schooling is estimated to be US$21.4 million.

**Table 2:** Cost of increasing coverage of nutrition interventions to 90% and cost of consequential increases in years of schooling, by socio-economic quintile.

Interventions	Cost of 90% coverage for birth cohort (Millions 2020 US$)					
	Q1	Q2	Q3	Q4	Q5	Total
Folic acid supplementation or fortification	3.90	3.11	2.63	2.78	2.30	14.73
Calcium supplementation in pregnancy	2.48	2.29	2.09	1.77	1.59	10.23
Multiple micronutrient supplementation in pregnancy (net of savings from reduced iron only supplementation in pregnancy)	1.08	1.01	0.93	0.81	0.72	4.55
Balanced energy supplementation	0.32	0.26	0.01	0.01	0	0.61
Breastfeeding promotion	4.67	4.36	4.06	3.45	3.15	19.69
Complementary feeding—supplementary feeding and education	6.07	3.93	0.17	0.16	0.01	10.34
Vitamin A Supplementation	0.75	0.60	0.49	0.31	0.13	2.29
Zinc supplementation	6.28	5.93	5.54	4.79	4.36	26.89
Therapeutic feeding for severe wasting (SAM)	0.18	0.03	0.12	0.09	0.01	0.43
Treatment for moderate acute malnutrition (MAM)	0.07	0.14	0.06	0.02	0.00	0.29
Total cost nutrition interventions	25.81	21.65	16.11	14.21	12.27	90.05
Cost of increased years of schooling arising from improved early nutrition	6.47	5.01	3.25	2.97	3.71	21.43
**Total cost**	32.28	26.67	19.36	17.19	15.99	111.48

Table 3 reports stunting rates at baseline and with increased coverage of the nutrition interventions and the implied improvement in mean HAZ and the number of lives saved, and the additional years of schooling that result from improved nutrition.

**Table 3:** Improvements in stunting at 24 months, mean HAZ-score, lives saved and additional years of schooling as a result of nutrition intervention scale up.

	Quintile					Total
	1	2	3	4	5	
Baseline stunting (%)	39.59	32.03	25.99	26.58	13.46	27.53
Post intervention stunting (%)	34.74	28	23.31	23.78	11.93	24.35
Percentage point decline in stunting	4.85	4.03	2.68	2.8	1.53	3.18
Improvement in mean HAZ-Score	0.13	0.12	0.09	0.09	0.07	0.10
Lives saved	920	460	460	0	0	1840
Additional years of schooling	13877	12538	9191	9495	7955	53055

The results suggest that if the nutrition interventions were scaled to 90% coverage, stunting rates among children at 24 months would decline by 3.18 percentage points overall, implying an increase in the mean HAZ of 0.10. Quintile 1 has the highest baseline rate of stunting, the largest decline as a result of intervention, and therefore the largest increase in mean HAZ.

Table 4 reports the estimated productivity returns associated with the 53000 years of school which occur as a result of scaling the nutrition interventions to 90% for three scenarios: (1) in the current context; (2) a context in which the gap in school quality is closed by 50%; (3) and a context with equitable school quality and equitable chances of finding employment.

**Table 4:** Productivity returns (Millions 2020 US$) and benefit cost ratios of scaling 10 early nutrition interventions, with and without changes to context.

	Quintile					Total
	1	2	3	4	5	
**Present value**						
Nutrition interventions only	506.39	431.67	4513.10	376.04	364.47	1979.44
Nutrition interventions in context of 50% school quality improvement	577.78	489.62	5103.00	413.81	364.47	2185.88
Nutrition interventions in context of equal school quality and labour market participation	987.32	831.87	8644.20	546.05	364.47	3305.99
**Cost benefit ratios: Value of returns per US$1 invested in nutrition interventions**						
Nutrition interventions only	16	16	16	22	23	18
Nutrition interventions in context of 50% school quality improvement	18	18	18	24	23	20
Nutrition interventions in context of common school quality and labour market participation	31	31	30	32	23	30

The results suggest scale up of nutrition interventions will yield productivity returns with a present value of close to US$2 billion. Quintile 1 would see the largest total productivity returns, at the highest cost. The cost benefit ratios imply that the highest return on investing in nutrition interventions in the current context would be seen in quintile 5 with a ratio of 1:23. The ratio implies that every US$1 invested in nutrition interventions for children in quintile 5 will yield benefits with a present value of US$23. Quintile 5 returns remain constant across scenarios as the scenarios relate to the impact of closing the gap in school quality and labour market participation between quintiles 1–4 and quintile 5. When inequality in school quality is reduced by half, quintile 4 yields almost the same return as quintile 5. If school quality and labour market opportunities are common across the quintiles, the highest return would be seen in quintile 1 (1:31).

The sensitivity analysis (S5 Appendix) highlights that the magnitude of returns is sensitive to several core assumptions. The finding that within the current environment there will be higher returns to investments in the early nutrition of children living in wealthier contexts is, however, not highly sensitive to the assumptions made. Investments in nutrition alone lead to the highest returns in quintile 1 only when we include the cost of delivery to everyone but only the benefits to children who would have been stunted.

### Limitations

The quintile models required combining data from multiple sources and the quintiles identified are unlikely to fully overlap. Estimating differences in returns to schooling associated with differences in school quality is challenging; and our estimates should be treated with caution. However, even if the quintile models are inadequate representations of the situation faced by children in those quintiles in South Africa, they still demonstrate the role of context.

To allow direct comparisons of total cost and total benefits across quintiles we assume that the cohort is evenly distributed between the quintiles. In South Africa, however, children are more likely to be in lower quintile households. As our headline results are ratios, distributing the children unevenly between quintiles would make no difference to our conclusion. We replicate the results in S5 Appendix with a more realistic but more difficult to interpret distribution of children by quintile, the ratios remain the same as the costs increase proportionally, we do not consider economies (or diseconomies) of scale or possible differences in cost of delivery of nutrition interventions between quintiles (we do consider differences in school costs). If it costs less to deliver to poorer children or there are significant diseconomies of scale, the cost benefit ratios of the lower quintiles would improve relative to quintile 5.

## Discussion

The returns to investments to support and enhance maternal and early childhood nutrition are high. In this South African case study we estimate that every US$1 invested in nutrition interventions would yield US$18 in productivity returns, as well as a range of other benefits. The ratio is towards the upper end of the range of existing estimates for sub-Saharan African countries: A World Bank estimate for scaling up the same interventions in 21 sub-Saharan African countries reported a ratio of 1:9 [38] while Hoddinott et al report ratios of between 1:4 (DRC) and 1:27 (Nigeria), again for the same interventions [39]. The ratio is, however, relatively low compared to estimates for countries in other low- and middle-income regions: 1:81 in South Asia [3]; between 1:19 (Middle East and North Africa) and 1:76 (East Asia and Pacific) reported by the World Bank [38].

Consider the different rates of return, to the same nutrition interventions within Africa and across other low- and middle-income countries. Read in the conventional manner, these results imply that the value of interventions to improve early nutrition is greater in South and East Asia than in Africa. Read considering the arguments presented here, these results suggest that a large proportion of the potential value of nutrition interventions in African countries will not be realised because of later life constraints on human development. The former interpretation justifies lower levels of investment in Africa. The latter calls for complementary interventions across the life course.

Children living in poorer households would benefit the most from nutrition interventions in terms of lives saved, improved nutrition outcomes and increased productivity. Average height at 2 years of age is projected to increase by 0.13 standard deviations for quintile 1 compared to 0.07 standard deviations for quintile 5. The estimated present value of productivity returns for quintile 1 is US$ 506.39 million compared to US$ 364.42 million in quintile 5. However the rate of return in quintile 1 is affected by the higher cost associated with reaching 90% coverage of interventions from a lower baseline coverage and greater need. Thus in quintile 1 the rate of return on investment is lower than in quintile 5: a benefit cost ratio of 1:16 compared to 1:23. This implies, counter-intuitively, that investing in childhood nutrition in the quintile with the best nutrition outcomes at baseline will yield the largest return in productivity over the life course. Moreover, the results imply that if the same amount were spent on improving nutrition outcomes in each quintile, inequality in lifetime earnings would increase. But these conclusions assume that the existing inequality in schooling and employment will continue. In other words, the productivity returns of investing in nutrition alone do not reflect the value of investing but rather the current distribution of life opportunities. While we estimate returns only in terms of productivity, the problem is likely to extend to other human development outcomes, given how important interactions with subsequent investments over the life course are, and how unequal these investments are.

The uneven distribution of returns can be ignored by reporting only the aggregate results. This can provide a powerful case for investment. Choosing to ignore the differences in returns across socio-economic profiles and reporting only aggregate results could be interpreted as an implicit statement by the analyst that they would never consider excluding lower socio-economic groups due to lower returns. They could similarly choose to ignore differences in returns across gender, race, disability and other factors that shape life chances and therefore returns. This effectively expands the evaluation space to include the type of society you want to see, in this case, one which does not discriminate on these grounds. Such an expansion can be justified [40], but should be explicit.

Alternatively, you can attach equity weights to the returns accruing to children in lower quintiles to reflect that similar increases may be more valuable to the poorest. Or you can focus only on the improvements in growth and lives saved, based on the assumption that outcomes should have the same value regardless of who benefits, i.e. prioritising based on cost effectiveness. But these approaches are problematic, because while they avoid discriminating against those in adversity, they still obscure differential returns and by so doing, miss a chance to question why the returns differ and what could be done to enable more equitable returns. By foregrounding the differential returns we highlight the implications of inequitable schooling and labour market opportunities, leading us to ask how these can be addressed if we are to maximise returns, and decrease inequality.

In South Africa poor quality education, as measured by learner performance, is linked to poor school management, limited parental involvement in children’s schooling, and ineffective assessment of learner capabilities [33,41]. Schools’ ability to efficiently utilize the resources allocated to them is linked to learner performance; thus, simply allocating greater resources to schools without effective management capacity will have limited impact [33]. Improving school quality requires a multipronged approach including management training, capacity development for teachers, improved supervision of schools by regional officials and efforts to increase parent involvement and oversight [33]. These efforts would need to focus on improving the quality of early education, particularly Grade R (reception year) and primary school, to limit the cumulative learning deficits [41].

Similarly, we need to consider how to improve labour market access for quintiles 1–4. In part, the unequal access to the labour market is a product of the unequal access to quality education. As a result, improving school quality would go some way to equalising employment rates. Another factor to be addressed is the major role of social networks, given how frequently job offers in South Africa are based on referrals from such networks [42]. The reliance on referrals is in part associated with widespread distrust among employers of official schooling results as a signal of productivity [43]. Recently, innovative interventions to improve how well labour force entrants can signal their capabilities to employers through standardised testing, have shown promise [43].

If we continue to disaggregate the returns to early nutrition interventions, we will see the possibility of increasing them in multiple ways. For example, in the model we only consider the difference in returns associated with school quality and the probability of finding employment, but children in quintile 5 receive a range of inputs, across their life course, which amplify the returns to education and therefore the benefits of early nutrition. Moreover, a disproportionate number in quintile 5 schools are white and the South African labour market discriminates in their favour. If we include higher returns associated with more inputs and a labour market that tends to favour white participants, the cost benefit ratio for investments in quintile 5 children raises from 1:23 to 1:35. The inputs which lead to differential returns are potential points of intervention, and the identification of higher rates of return on investments in white children highlights the need to address systematic racism in the labour market.

Disaggregating returns and identifying what may be limiting the realisation and utilisation of the human potential that early nutrition interventions protect is not the same as a joint evaluation of nutrition interventions plus later life interventions. Joint evaluations would require us to consider the costs of all the interventions and all the benefits, which we do not. Our goal is to highlight that for children living in adversity early nutrition interventions protect potential human development that is not wholly realised and utilised later in life. This is because returns to early nutrition interventions are sensitive to context, and can thus be increased by reasonable improvements in context. Therefore if we do not identify and address the contextual constraints on the realisation and utilisation of potential, we squander some of the gains of early intervention. The more adverse the environment, the higher the proportion of gains squandered. A failure to identify and weaken constraints will lead not only to nutrition interventions being undervalued in priority setting but will deny the children living in adversity the opportunity to fully benefit from them even when they are received. The argument holds for a range of interventions in early life.

We have focused on a country case study, but the implications for cross-country comparisons are clear. It is near impossible for a country to justify prioritising services to wealthier children based on higher returns. But prioritising services for children in wealthier countries is common practice. Priority setting approaches which apply a GDP based threshold, for example, imply that certain services should only be offered to children in wealthier countries where the returns will be higher [44–46]. The difference is that within a country you have the same duty holder and across countries you have different duty holders i.e. different governments. This may justify different thresholds based on affordability, but this should never be confused with differences in value. Valued in terms of protected potential benefits, early life interventions would have similar rates of return across countries. It is only when they are valued in terms of realised and utilised benefits that the differences emerge, reflecting patterns of inequality. If we are interested in maximising value and minimising inequality in human development outcomes, low returns in terms of realised or utilised benefits should trigger more intervention not less. The problem is that low returns and tighter financial constraints go hand in hand. Inequality replicates itself.

## Conclusion

Ensuring adequate nutrition for a child who faces abuse, poor quality schooling, and an environment which curtails their rights in adulthood will prevent them from being hungry in childhood, but without other interventions they will not realise the full benefits that improved nutrition can allow. To see the full benefit of improved early nutrition, especially when children face numerous challenges, we must avoid focusing on nutrition alone and rather ask what is required so that children can realise and utilise their protected potential. We have focused on productivity returns, but the conclusions apply to other human development outcomes. Indeed, we would support a broadly defined evaluation space which includes considering the role of complementary interventions across the life course in promoting a range of human development outcomes, reflecting what people value. and who they want to be [47,48].

## Supporting information

S1 Appendix Projection baseline parameters. (DOC)

S2 Appendix Costing methods. (DOC)

S3 Appendix Projected stunting rate. (DOC)

S4 Appendix Assumptions for productivity estimates. (DOC)

S5 Appendix Sensitivity analysis. (DOC)

S6 Appendix Supplementary material references. (DOC)
