# Supplementary material for: Realising the potential human development returns to investing in early and maternal nutrition: The importance of identifying and addressing constraints over the life course
Source: PLOS Glob Public Health. 2021 Oct 13;1(10):e0000021. doi: 10.1371/journal.pgph.0000021 (PMC10022083; doi:10.1371/journal.pgph.0000021)
Supplement: S1 Appendix — (DOC) [file pgph.0000021.s001.doc]

# **S1 Appendices 1-6**

## **S1 appendix 1: projection baseline parametres**

### **DEMPROJ**

All parameters not specified here are default DemProj values for South Africa.

| First year | 2020 |
| --- | --- |
| Final year | 2025 |
| First year population by single year of age 0-80 | Default values multiplied by 0.2 for each quintile |
| Sex ratio at birth | SADHS 2016 overall sex ratio at birth, living births average 2012-2016 = 105.1 duplicated for all future projection years.^1^ |

|  | **Q1** | **Q2** | **Q3** | **Q4** | **Q5** |  |
| --- | --- | --- | --- | --- | --- | --- |
| Total fertility rate | 3.1 | 2.9 | 2.7 | 2.3 | 2.1 | SADHS 2016, 2020 values duplicated to 2025.^1^ |

**AIM**

All parameters not specified here are default AIM values for South Africa.

| Child treatment | UNAIDS estimate for % receiving ART=63% duplicated across years, no data by age, % lost to follow-up^3^ |
| --- | --- |
| Adult ART | 65% of women and 56% of men living with HIV receiving ART ^3^ |
| Incidence | National value of 4.94 duplicated across years^3^ |

**LiST**

All parameters not specified here are default LiST values for South Africa. The full sources for LiST default values can be found within the LiST module.

| **Health and well being** | **Q1** | **Q2** | **Q3** | **Q4** | **Q5** | **Source** |
| --- | --- | --- | --- | --- | --- | --- |
| Term: Small for gestational age SGA is defined as <10th percentile (defined as weighing less than 2.5 kg at birth). | 15.4 | 16.3 | 13.1 | 15.5 | 12.9 | Authors’ calculation from SADHS 2016 data.^1^ |
| % pregnant women with anemia  (hemoglobin levels < 110 g/L.) | 35.3 | 37.1 | 56.3 | 31.9 | 29.0 | Authors’ calculation from SADHS 2016 data.^1^ |
| % non-pregnant women age 15-49 with anemia (hemoglobin levels < 110 g/L.) | 30.4 | 36.0 | 32.5 | 35.3 | 32.0 |
| % women with low BMI aged 15-49 with BMI <18.5 | 2.6 | 3.3 | 4.0 | 2.4 | 2.5 |
| Stunting percentage Below -2 SD | 36.3 | 29.4 | 23.9 | 24.5 | 12.5 | SADHS 2016.^2^ |
| Wasting percentage Below -2 SD | 2.0 | 4.1 | 2.6 | 1.8 | 0.1 |
| Under-5 Deaths per 1,000 live births for the 10-year period before the survey | 67 | 52 | 51 | 34 | 41 |
| Infant per 1,000 live births for the 10-year period before the survey | 54 | 44 | 46 | 27 | 40 |
| Early neonatal mortality (0-6 days among live-born children) rate for the 5-year period preceding the survey*  *Spectrum requires 28 days | 20 | 27 | 9 | 7 | 4 |
| Still birth rate per 1000 live births rate for the 5-year period preceding the survey | 9 | 10 | 8 | 5 | 4 |
| Percentage of children aged 7-17 living below the poverty line and household income per capita quintile 2015 | 83.9 | 57.1 | 2.8 | 4.6 | 0.5 | Statistics South Africa, 2019.^4^ |
| Average household size 2011 | 5.8 | 4.3 | 3.5 | 3.0 | 2.6 | Statistics South Africa, 2015.^5^(Duplicated until 2025) |

| **Intervention coverage** | **Q1** | **Q2** | **Q3** | **Q4** | **Q5** | **Source** |
| --- | --- | --- | --- | --- | --- | --- |
| Folic acid supplementation | 72 | 77 | 80 | 79 | 82 | Authors’ calculation from SADHS 2016 data and expert consultation.^6^ |
| Percentage of women who took iron tablets during the pregnancy of their most recent live birth | 87.7 | 90.9 | 90.5 | 92.6 | 87.8 | SADHS 2016.^2^ |
| Percentage whose most recent live birth was protected against neonatal tetanus | 35.4 | 34.7 | 35.8 | 37.3 | 31.7 |
| Pregnancy: Calcium supplementation | 18 | 19 | 20 | 20 | 21 | Authors’ calculation from SADHS 2016 data and expert consultation.^6^ |
| Skilled birth attendance (percentage of live births delivered by skilled attendant) | 92.6 | 96.3 | 98.4 | 98.5 | 98.9 | SADHS 2016.^2^ |
| Health facility delivery (percent of live births delivered in health facility)  ** skilled birth attendance must be greater or equal to health facility delivery therefore this value has been entered as 98.4* | 91.6 | 95.2 | 98.2 | 96.9 | 99.4* |
| Prevalence of early initiation of breastfeeding  Among last-born children born in the past 2 years: Percentage who started breastfeeding within 1 hour of birth | 67.4 | 67.2 | 69.2 | 64.2 | 68.8 | SADHS 2016.^2^ |
| Percentage of children less than six months of age who are being exclusively breastfed | 31.6 | 29.8 | 47.9 | 31.7 | 23.3 | Countdown to 2030.^7^ |
| Exclusive breastfeeding <1mo | 47.4 | 44.7 | 71.85 | 47.55 | 34.95 | Authors’ calculation from SADHS 2016 data.^2^ |
| Continued breastfeeding (year 1) | 51.4 | 62.3 | 41.6 | 44.2 | 59.9 | SADHS 2016.^2^ |
| Among all children age 6-59 months: Percentage given vitamin A supplements in past 6 months | 70.3 | 73.3 | 73.3 | 70.6 | 74.3 |
| Proportion of households with access to improved sanitation by per capita income quintile 2015 | 69.2 | 74.6 | 81.4 | 89.6 | 96.2 | Statistics South Africa, 2019.^8^ |
| Improved water source | 65.5 | 88.8 | 93.1 | 94.5 | 99.5 | World Bank, 2015^9^ |
| Proportion of households with access to piped or tap water inside the dwelling or on-site by income per-capita quintile | 56.6 | 70.6 | 81.6 | 89.0 | 93.9 | Statistics South Africa, 2019.^8^ |
| Among households in which place for handwashing was observed, percentage with: Soap and water | 23.0 | 30.4 | 38.0 | 61.6 | 86.7 | SADHS 2016.^2^ |
| Complementary feeding - supplementary feeding and education  Proxy used: Among all children 6-23 months, percentage fed minimum dietary diversity | 46.7 | 43.6 | 44.9 | 55.7 | 62.0 |
| BCG | 89.1 | 94.3 | 92.5 | 96.6 | 89.7 | Authors’ calculation from SADHS 2016.^2^ |
| Polio | 83.6 | 69 | 79 | 88.2 | 66.2 |
| DPT | 72 | 54.8 | 68.4 | 67.8 | 62.2 |
| Hib | 72 | 54.8 | 68.4 | 67.8 | 62.2 |
| Hep B | 71 | 58.7 | 65.9 | 67 | 61.5 |
| Pneumococcal | 68.2 | 54.5 | 65.3 | 61.1 | 60.3 |
| Rotavirus | 74.1 | 64 | 70.4 | 76.6 | 65 |
| Malaria | 0 | 0 | 0 | 0 | 0 |
| Measles | 85.1 | 84.6 | 87.5 | 87 | 87.6 |
| Percentage of children with diarrhea who received oral rehydration salts | 51.4 | 39.6 | 67.2 | 64.2 | 68.8 | Countdown to 2030.^7^ |
| MAM | 40 | 43 | 45 | 44 | 46 | Authors’ calculation from SADHS 2016 data and expert consultation.^6^ |
| SAM | 22 | 24 | 25 | 25 | 26 |

| **Quintile definitions** |
| --- |
| National wealth quintiles compiled by assigning the household score to each usual (de jure) household member, ranking each person in the household population by her or his score, and then dividing the distribution into five equal categories, each comprising 20% of the population.^2^ |
| Per capita income quintile.^4^ |
| Households divided into expenditure quintiles according to per capita expenditure data. ^5^ |
| Per capita income quintile^8^ |
| Undefined^9^ |
