# Supplementary material for: Realising the potential human development returns to investing in early and maternal nutrition: The importance of identifying and addressing constraints over the life course
Source: PLOS Glob Public Health. 2021 Oct 13;1(10):e0000021. doi: 10.1371/journal.pgph.0000021 (PMC10022083; doi:10.1371/journal.pgph.0000021)
Supplement: S2 Appendix — (DOC) [file pgph.0000021.s002.doc]

**S1 appendix 2: costing methods**

### **1. Staff baseline cost**

| **Custom Annual Salary (US$)** | | | | |
| --- | --- | --- | --- | --- |
| **LiST Intervention staff** | **DPSA Equivalent position (Full time)**^9^ | **2018**^9^ | **2019**  ***(3% CPI^*^ adjusted from 2018)*** | **2020**  ***(3% CPI^*^ adjusted from 2019)*** |
| Generalists/primary care doctors | TCE: Medical Specialist Gr 1 | 70091-74392 | 74409 | 76641 |
| Ob\Gyns | TCE: Medical Specialist (sub specialty) Gr 1 | 81344-86335 | 86354 | 88945 |
| Pediatricians | TCE: Medical Specialist (sub specialty) Gr 1 | 81344-86335 | 86354 | 88945 |
| Other specialist doctors | TCE: Medical Specialist (sub specialty) Gr 1 | 81344-86335 | 86354 | 88945 |
| Clinical officers/surgical technicians | TCE: Medical Specialist Gr 1 | 70091-74392 | 74409 | 76641 |
| Nurses | Notch: Professional nurse | 13231 | 13628 | 14037 |
| Midwives | Professional Nurse Gr 1 (Specialty Nursing) | 24171-28021 | 26879 | 27685 |
| Assistant nurses and midwives | Notch: Nursing Assistant Gr 1 | 8319-9364 | 9107 | 9380 |
| Nursing aides | Notch: Nursing Assistant Gr 1 | 8319-9364 | 9107 | 9380 |
| Laboratory technicians/assistants | Notch: Full time Radiation Laboratory Technician Gr 2 | 18895 | 19462 | 20046 |
| Pharmaceutical technicians/ assistants | Notch: Full time Pharmacist Assistant (Basic) Gr 1 | 7721-8195 | 8197 | 8443 |
| Radiographers/X-ray technicians | Notch: Diagnostic Radiographer Gr 1 | 22824 | 23509 | 24214 |
| Emergency medical technicians | Notch: Emergency Care Technician Gr 1 | 13081-15640 | 14792 | 15235 |
| Community health workers | Notch: Assistant Community Development Practitioner Gr 1 | 8849-9871 | 9641 | 9930 |
| Other |  |  |  | 7706^ |

^ Default LiST value.

*Annual consumer price index inflation was 3.0% in April 2020^10^

### **2. Target population**

| **Intervention** | **Target population** |
| --- | --- |
| Folic acid supplementation/fortification | Women of reproductive age (15-49)* |
| Calcium supplementation | Pregnant women* |
| Iron supplementation in pregnancy |
| Multiple micronutrient supplementation in pregnancy |
| Balanced energy supplementation |
| Promotion of breastfeeding | Live births* |
| Complementary feeding - supplementary feeding and education | Children 6-23 months^#^ |
| Vitamin A supplementation |
| Zinc supplementation |
| SAM - treatment for severe acute malnutrition |
| MAM - treatment for moderate acute malnutrition |

^*^ LiST Default.

^#^ Authors’ own.

### **3. Population in need (disease incidence) %**

Population in need is determined by incidence and prevalence of conditions, as well as by treatment guidelines.

Default values automatically adjusted by LiST per quintile model

| **QUINTILE 1** | | | | | | |
| --- | --- | --- | --- | --- | --- | --- |
| **Intervention** | **2020** | **2021** | **2022** | **2023** | **2024** | **2025** |
| Folic acid supplementation/fortification | 100 | 100 | 100 | 100 | 100 | 100 |
| Calcium supplementation | 100 | 100 | 100 | 100 | 100 | 100 |
| Micronutrient supplementation (iron and multiple micronutrients) | 100 | 100 | 100 | 100 | 100 | 100 |
| Balanced energy supplementation | 2.18 | 2.18 | 2.18 | 2.18 | 2.18 | 2.18 |
| Promotion of breastfeeding | 100 | 100 | 100 | 100 | 100 | 100 |
| Complementary feeding - supplementary feeding and education* | 83.9 | 83.9 | 83.9 | 83.9 | 83.9 | 83.9 |
| Vitamin A supplementation | 100 | 100 | 100 | 100 | 100 | 100 |
| Zinc supplementation | 100 | 100 | 100 | 100 | 100 | 100 |
| SAM - treatment for severe acute malnutrition | 0.12 | 0.12 | 0.12 | 0.12 | 0.12 | 0.12 |
| MAM - treatment for moderate acute malnutrition | 1.88 | 1.88 | 1.88 | 1.88 | 1.88 | 1.88 |

*The original assumption for complementary feeding - supplementary feeding and education uses the proxy: “Among all children 6-23 months, percentage fed minimum dietary diversity”

| **QUINTILE 2** | | | | | | |
| --- | --- | --- | --- | --- | --- | --- |
| **Intervention** | **2020** | **2021** | **2022** | **2023** | **2024** | **2025** |
| Folic acid supplementation/fortification | 100 | 100 | 100 | 100 | 100 | 100 |
| Calcium supplementation | 100 | 100 | 100 | 100 | 100 | 100 |
| Micronutrient supplementation (iron and multiple micronutrients) | 100 | 100 | 100 | 100 | 100 | 100 |
| Balanced energy supplementation | 1.9 | 1.9 | 1.9 | 1.9 | 1.9 | 1.9 |
| Promotion of breastfeeding | 100 | 100 | 100 | 100 | 100 | 100 |
| Complementary feeding - supplementary feeding and education | 57.1 | 57.1 | 57.1 | 57.1 | 57.1 | 57.1 |
| Vitamin A supplementation | 100 | 100 | 100 | 100 | 100 | 100 |
| Zinc supplementation | 100 | 100 | 100 | 100 | 100 | 100 |
| SAM - treatment for severe acute malnutrition | 1.2 | 0.5 | 0.5 | 0.5 | 0.4 | 0.4 |
| MAM - treatment for moderate acute malnutrition | 3.0 | 1.3 | 1.3 | 1.3 | 1.3 | 1.3 |

| **QUINTILE 3** | | | | | | |
| --- | --- | --- | --- | --- | --- | --- |
| **Intervention** | **2020** | **2021** | **2022** | **2023** | **2024** | **2025** |
| Folic acid supplementation/fortification | 100 | 100 | 100 | 100 | 100 | 100 |
| Calcium supplementation | 100 | 100 | 100 | 100 | 100 | 100 |
| Micronutrient supplementation (iron and multiple micronutrients) | 100 | 100 | 100 | 100 | 100 | 100 |
| Balanced energy supplementation | 0.1 | 0.1 | 0.1 | 0.1 | 0.1 | 0.1 |
| Promotion of breastfeeding | 100 | 100 | 100 | 100 | 100 | 100 |
| Complementary feeding - supplementary feeding and education | 2.8 | 2.8 | 2.8 | 2.8 | 2.8 | 2.8 |
| Vitamin A supplementation | 100 | 100 | 100 | 100 | 100 | 100 |
| Zinc supplementation | 100 | 100 | 100 | 100 | 100 | 100 |
| SAM - treatment for severe acute malnutrition | 0.3 | 0.2 | 0.2 | 0.2 | 0.2 | 0.2 |
| MAM - treatment for moderate acute malnutrition | 2.3 | 0.9 | 0.9 | 0.9 | 0.9 | 0.8 |

| **QUINTILE 4** | | | | | | |
| --- | --- | --- | --- | --- | --- | --- |
| **Intervention** | **2020** | **2021** | **2022** | **2023** | **2024** | **2025** |
| Folic acid supplementation/fortification | 100 | 100 | 100 | 100 | 100 | 100 |
| Calcium supplementation | 100 | 100 | 100 | 100 | 100 | 100 |
| Micronutrient supplementation (iron and multiple micronutrients) | 100 | 100 | 100 | 100 | 100 | 100 |
| Balanced energy supplementation | 0.1 | 0.1 | 0.1 | 0.1 | 0.1 | 0.1 |
| Promotion of breastfeeding | 100 | 100 | 100 | 100 | 100 | 100 |
| Complementary feeding - supplementary feeding and education | 4.6 | 4.6 | 4.6 | 4.6 | 4.6 | 4.6 |
| Vitamin A supplementation | 100 | 100 | 100 | 100 | 100 | 100 |
| Zinc supplementation | 100 | 100 | 100 | 100 | 100 | 100 |
| SAM - treatment for severe acute malnutrition | 0.1 | 0.1 | 0.1 | 0.1 | 0.1 | 0.1 |
| MAM - treatment for moderate acute malnutrition | 1.7 | 0.6 | 0.6 | 0.6 | 0.6 | 0.6 |

| **QUINTILE 5** | | | | | | |
| --- | --- | --- | --- | --- | --- | --- |
| **Intervention** | **2020** | **2021** | **2022** | **2023** | **2024** | **2025** |
| Folic acid supplementation/fortification | 100 | 100 | 100 | 100 | 100 | 100 |
| Calcium supplementation | 100 | 100 | 100 | 100 | 100 | 100 |
| Micronutrient supplementation (iron and multiple micronutrients) | 100 | 100 | 100 | 100 | 100 | 100 |
| Balanced energy supplementation | 0.01 | 0.01 | 0.01 | 0.01 | 0.01 | 0.01 |
| Promotion of breastfeeding | 100 | 100 | 100 | 100 | 100 | 100 |
| Complementary feeding - supplementary feeding and education | 0.50 | 0.50 | 0.50 | 0.50 | 0.50 | 0.50 |
| Vitamin A supplementation | 100 | 100 | 100 | 100 | 100 | 100 |
| Zinc supplementation | 100 | 100 | 100 | 100 | 100 | 100 |
| SAM - treatment for severe acute malnutrition | 0.00 | 0.00 | 0.00 | 0.00 | 0.00 | 0.00 |
| MAM - treatment for moderate acute malnutrition | 0.01 | 0.00 | 0.00 | 0.00 | 0.00 | 0.00 |

### **4. Treatment inputs**

Default LiST values used.

### **5. Other-recurrent and capital costs**

Default LiST values used.

### **6. Delivery channels**

Default LiST values used.

### **7. Program costs**

Default LiST values used.

### **8. Logistics and wastage**

Default LiST values used.

### **9. Infrastructure investment**

Default LiST values used.

### **10. Other health system costs**

Default LiST values used.

### **11. Inefficiencies**

Default LiST values used.

### **12. Configuration- currency and inflation**

|  | **2020** | **2021** | **2022** | **2023** | **2024** | **2025** |
| --- | --- | --- | --- | --- | --- | --- |
| Exchange rate (ZAR-USD) | 15.00 | 15.00 | 15.00 | 15.00 | 15.00 | 15.00 |
| Domestic inflation rate | 0 | 0 | 0 | 0 | 0 | 0 |
| USD inflation rate | 0 | 0 | 0 | 0 | 0 | 0 |
