# Supplementary material for: Realising the potential human development returns to investing in early and maternal nutrition: The importance of identifying and addressing constraints over the life course
Source: PLOS Glob Public Health. 2021 Oct 13;1(10):e0000021. doi: 10.1371/journal.pgph.0000021 (PMC10022083; doi:10.1371/journal.pgph.0000021)
Supplement: S3 Appendix — (DOC) [file pgph.0000021.s003.doc]

## **S1 appendix 3: projected stunting rate**

| **Age group** | **QUINTILE 1 BASELINE Global stunting (<-2 SD) rate** | | | | | | **QUINTILE 1 SCENARIO 1 Global stunting (<-2 SD) rate** | | | | | |
| --- | --- | --- | --- | --- | --- | --- | --- | --- | --- | --- | --- | --- |
|  | **2020** | **2021** | **2022** | **2023** | **2024** | **2025** | **2020** | **2021** | **2022** | **2023** | **2024** | **2025** |
| <1 month | 17.53 | 17.53 | 17.53 | 17.53 | 17.53 | 17.53 | 17.53 | 16.86 | 16.86 | 16.86 | 16.86 | 16.86 |
| 1-5 months | 17.53 | 17.53 | 17.53 | 17.53 | 17.53 | 17.53 | 17.53 | 16.75 | 16.75 | 16.75 | 16.75 | 16.75 |
| 6-11 months | 22.24 | 22.24 | 22.24 | 22.24 | 22.24 | 22.24 | 22.24 | 20.59 | 20.59 | 20.59 | 20.59 | 20.59 |
| 12-23 months | 39.59 | 39.59 | 39.59 | 39.59 | 39.59 | 39.59 | 39.59 | 35.87 | 34.74 | 34.74 | 34.74 | 34.74 |
| 24-59 months | 40.67 | 40.67 | 40.67 | 40.67 | 40.67 | 40.67 | 40.67 | 38.15 | 37.14 | 35.84 | 34.53 | 34.23 |
| Total (0-59 months) | 35.31 | 35.23 | 35.42 | 35.65 | 35.88 | 36.09 | 35.31 | 32.82 | 32.15 | 31.65 | 31.1 | 31.07 |

| **Age group** | **QUINTILE 2 BASELINE Global stunting (<-2 SD) rate** | | | | | | **QUINTILE 2 SCENARIO 1 Global stunting (<-2 SD) rate** | | | | | |
| --- | --- | --- | --- | --- | --- | --- | --- | --- | --- | --- | --- | --- |
|  | **2020** | **2021** | **2022** | **2023** | **2024** | **2025** | **2020** | **2021** | **2022** | **2023** | **2024** | **2025** |
| <1 month | 14.43 | 14.43 | 14.43 | 14.43 | 14.43 | 14.43 | 14.43 | 13.89 | 13.89 | 13.89 | 13.89 | 13.89 |
| 1-5 months | 14.43 | 14.43 | 14.43 | 14.43 | 14.43 | 14.43 | 14.43 | 13.84 | 13.84 | 13.84 | 13.84 | 13.84 |
| 6-11 months | 18.19 | 18.19 | 18.19 | 18.19 | 18.19 | 18.19 | 18.19 | 16.97 | 16.97 | 16.97 | 16.97 | 16.97 |
| 12-23 months | 32.03 | 32.03 | 32.03 | 32.03 | 32.03 | 32.03 | 32.03 | 28.88 | 28 | 28 | 28 | 28 |
| 24-59 months | 32.89 | 32.89 | 32.89 | 32.89 | 32.89 | 32.89 | 32.89 | 30.6 | 29.77 | 28.69 | 27.63 | 27.39 |
| Total (0-59 months) | 28.8 | 28.76 | 28.87 | 29.01 | 29.14 | 29.28 | 28.8 | 26.64 | 26.06 | 25.58 | 25.08 | 25.03 |

| **Age group** | **QUINTILE 3 BASELINE Global stunting (<-2 SD) rate** | | | | | | **QUINTILE 3 SCENARIO 1 Global stunting (<-2 SD) rate** | | | | | |
| --- | --- | --- | --- | --- | --- | --- | --- | --- | --- | --- | --- | --- |
|  | **2020** | **2021** | **2022** | **2023** | **2024** | **2025** | **2020** | **2021** | **2022** | **2023** | **2024** | **2025** |
| <1 month | 11.97 | 11.97 | 11.97 | 11.97 | 11.97 | 11.97 | 11.97 | 11.64 | 11.64 | 11.64 | 11.64 | 11.64 |
| 1-5 months | 11.97 | 11.97 | 11.97 | 11.97 | 11.97 | 11.97 | 11.97 | 11.51 | 11.51 | 11.51 | 11.51 | 11.51 |
| 6-11 months | 14.97 | 14.97 | 14.97 | 14.97 | 14.97 | 14.97 | 14.97 | 14.35 | 14.35 | 14.35 | 14.35 | 14.35 |
| 12-23 months | 25.99 | 25.99 | 25.99 | 25.99 | 25.99 | 25.99 | 25.99 | 23.77 | 23.31 | 23.31 | 23.31 | 23.31 |
| 24-59 months | 26.68 | 26.68 | 26.68 | 26.68 | 26.68 | 26.68 | 26.68 | 24.67 | 24.09 | 23.38 | 22.68 | 22.56 |
| Total (0-59 months) | 23.58 | 23.55 | 23.6 | 23.67 | 23.74 | 23.81 | 23.58 | 21.84 | 21.45 | 21.1 | 20.74 | 20.72 |

| **Age group** | **QUINTILE 4 BASELINE Global stunting (<-2 SD) rate** | | | | | | **QUINTILE 4 SCENARIO 1 Global stunting (<-2 SD) rate** | | | | | |
| --- | --- | --- | --- | --- | --- | --- | --- | --- | --- | --- | --- | --- |
|  | **2020** | **2021** | **2022** | **2023** | **2024** | **2025** | **2020** | **2021** | **2022** | **2023** | **2024** | **2025** |
| <1 month | 12.66 | 12.66 | 12.66 | 12.66 | 12.66 | 12.66 | 12.66 | 12.29 | 12.29 | 12.29 | 12.29 | 12.29 |
| 1-5 months | 12.66 | 12.66 | 12.66 | 12.66 | 12.66 | 12.66 | 12.66 | 12.15 | 12.15 | 12.15 | 12.15 | 12.15 |
| 6-11 months | 15.64 | 15.64 | 15.64 | 15.64 | 15.64 | 15.64 | 15.64 | 14.96 | 14.96 | 14.96 | 14.96 | 14.96 |
| 12-23 months | 26.58 | 26.58 | 26.58 | 26.58 | 26.58 | 26.58 | 26.58 | 24.3 | 23.78 | 23.78 | 23.78 | 23.78 |
| 24-59 months | 27.26 | 27.26 | 27.26 | 27.26 | 27.26 | 27.26 | 27.26 | 25.21 | 24.61 | 23.88 | 23.15 | 23.02 |
| Total (0-59 months) | 24.52 | 24.51 | 24.49 | 24.47 | 24.46 | 24.45 | 24.52 | 22.71 | 22.23 | 21.78 | 21.33 | 21.25 |

| **Age group** | **QUINTILE 5 BASELINE Global stunting (<-2 SD) rate** | | | | | | **QUINTILE 5 SCENARIO 1 Global stunting (<-2 SD) rate** | | | | | |
| --- | --- | --- | --- | --- | --- | --- | --- | --- | --- | --- | --- | --- |
|  | **2020** | **2021** | **2022** | **2023** | **2024** | **2025** | **2020** | **2021** | **2022** | **2023** | **2024** | **2025** |
| <1 month | 7.05 | 7.05 | 7.05 | 7.05 | 7.05 | 7.05 | 7.05 | 6.85 | 6.85 | 6.85 | 6.85 | 6.85 |
| 1-5 months | 7.05 | 7.05 | 7.05 | 7.05 | 7.05 | 7.05 | 7.05 | 6.86 | 6.86 | 6.86 | 6.86 | 6.86 |
| 6-11 months | 8.42 | 8.42 | 8.42 | 8.42 | 8.42 | 8.42 | 8.42 | 8.14 | 8.14 | 8.14 | 8.14 | 8.14 |
| 12-23 months | 13.46 | 13.46 | 13.46 | 13.46 | 13.46 | 13.46 | 13.46 | 12.14 | 11.93 | 11.93 | 11.93 | 11.93 |
| 24-59 months | 13.77 | 13.77 | 13.77 | 13.77 | 13.77 | 13.77 | 13.77 | 12.57 | 12.24 | 11.87 | 11.49 | 11.44 |
| Total (0-59 months) | 12.59 | 12.59 | 12.56 | 12.53 | 12.5 | 12.47 | 12.59 | 11.54 | 11.28 | 11.03 | 10.78 | 10.73 |
