# Supplementary material for: Realising the potential human development returns to investing in early and maternal nutrition: The importance of identifying and addressing constraints over the life course
Source: PLOS Glob Public Health. 2021 Oct 13;1(10):e0000021. doi: 10.1371/journal.pgph.0000021 (PMC10022083; doi:10.1371/journal.pgph.0000021)
Supplement: S4 Appendix — (DOC) [file pgph.0000021.s004.doc]

## **S1 appendix 4: assumptions for productivity estimates**

**Model assumptions**

| **Variable** | **Value** | **Source** | **Notes** |
| --- | --- | --- | --- |
| Increase in years of schooling per Standard Deviation increase in Z-Score (IYS) | 0.47 | Fink *et al*, 2016.^12^ | Based on analysis reported in Adair et al, 2013.^13^ |
| Proxy for returns to education for quintile 2/3 (P_2,3_) | 15.9% | Salisbury, 2016.^14^ | Estimate of returns to education for Black South Africans. |
| Proxy for returns to education for quintile 5 (P_5_) | 22.6% | Salisbury, 2016.^14^ | Estimate of returns to education for White South Africans |
| Proportion of difference in returns to education explained by differences in quality of schooling (RD) | 0.37 | Van Der Berg *et al*, 2011.^15^ | Balance of variation in returns associated with differences in other inputs correlated with wealth and systemic racism. |
| Proxy of school quality by quintile, matric bachelor pass rate by school quintile (1-5) (SQq) | 0.37; 0.39; 0.40; 0.45; 0.61 | DBE, 2020.^16^ |  |
| Average years of schooling without intervention, by quintile (1-5) (AYSq) | 10; 10.5; 10.9; 11.1; 11.5. | Author calculations using GHS, 2018.^17^ | Average years of education for respondents age 20-25 years by asset index quintile |
| Years until entry in to the labour force (YLF) | 20 | Alderman *et al*, 2017.^18^ |  |
| Annual wage with zero schooling (AW) | R21,000 | Salisbury, 2016.^14^ | 2008 estimate adjusted to 2020 using SA CPI.^10^ |
| Baseline labour force participation rate by quintile (1-5) (LFPq) | 0.4; 0.4; 0.4; 0.5; 0.6 | Author calculations using GHS, 2018.^17^ | Labour force participation rate by asset index for respondents 20-60 years of age |
| Working life (years) (WY) | 40 | Alderman *et a*l, 2017.^18^ |  |
| Discount rate (DR) | 3% | Assumption |  |
| Annual growth rate in real income (IG) | 1% | Assumption |  |

| **Key calculations** | **Methods/formula** |
| --- | --- |
| RTEqs = Returns to education (quintile and scenario) | RTE_5_s = P_2,3_ + (P_5_-P_2,3_)*RD  RTEq_0_ = RTEq_1_ = C + B*(SQq)  RTEq_2_ = RTEq_1_ + (0.5 * (RTE_5_s - RTEq_0_)  RTEq_3_ = RTE_5_s  Where:  B = (RTE_5_s – P_2,3_)/(SQ_5_-(SQ_2_+SQ_3_)/2)  C = RTE_5_s – (SQ_5_*B) |
| Present value of life time income (quintile/scenario) | LIqs = Sum (n=1-WY) [Present Value (AW * (1+RTEqs)^AYSqs)*(1+IG)^YLF)*(1+IG)^n)*LFPqs] |
