# Supplementary material for: Realising the potential human development returns to investing in early and maternal nutrition: The importance of identifying and addressing constraints over the life course
Source: PLOS Glob Public Health. 2021 Oct 13;1(10):e0000021. doi: 10.1371/journal.pgph.0000021 (PMC10022083; doi:10.1371/journal.pgph.0000021)
Supplement: S5 Appendix — (DOC) [file pgph.0000021.s005.doc]

## **S1 appendix 5: sensitivity analysis**

| **Baseline** | **Q1** | **Q5** |
| --- | --- | --- |
| Nutrition interventions only | 16 | 23 |
| Nutrition interventions in context of school improvement | 18 | 23 |
| Nutrition interventions in context of common school quality and labour market opportunities | 31 | 23 |
| **High estimate of impact on stunting (LiST Sensitivity Analysis)** | | |
| Nutrition interventions only | 24 | 38 |
| Nutrition interventions in context of school improvement | 28 | 38 |
| Nutrition interventions in context of common school quality and labour market opportunities | 47 | 38 |
| **Low estimate of impact on stunting (LiST Sensitivity Analysis)** | | |
| Nutrition interventions only | 9 | 9 |
| Nutrition interventions in context of school improvement | 10 | 9 |
| Nutrition interventions in context of common school quality and labour market opportunities | 18 | 9 |
| **Benefits only to children who would have been stunted in the absence of additional intervention** | | |
| Nutrition interventions only | 6 | 3 |
| Nutrition interventions in context of school improvement | 7 | 3 |
| Nutrition interventions in context of common school quality and labour market opportunities | 12 | 3 |
| **Smaller variance in school returns explained by quality (-10 percentage points)** | | |
| Nutrition interventions only | 16 | 21 |
| Nutrition interventions in context of school improvement | 17 | 21 |
| Nutrition interventions in context of common school quality and labour market opportunities | 29 | 21 |
| **Larger variance in school returns explained by quality (+10 percentage points)** | | |
| Nutrition interventions only | 16 | 24 |
| Nutrition interventions in context of school improvement | 18 | 24 |
| Nutrition interventions in context of common school quality and labour market opportunities | 33 | 24 |
| **Larger impact of growth on years of completed schooling (+50%)** | | |
| Nutrition interventions only | 21 | 31 |
| Nutrition interventions in context of school improvement | 24 | 31 |
| Nutrition interventions in context of common school quality and labour market opportunities | 42 | 31 |
| **Smaller impact of growth on years of completed schooling (-50%)** | | |
| Nutrition interventions only | 9 | 13 |
| Nutrition interventions in context of school improvement | 10 | 13 |
| Nutrition interventions in context of common school quality and labour market opportunities | 17 | 13 |
| **Discount rate 5%** | | |
| Nutrition interventions only | 8 | 11 |
| Nutrition interventions in context of school improvement | 9 | 11 |
| Nutrition interventions in context of common school quality and labour market opportunities | 15 | 11 |
| **Discount rate 0%** | | |
| Nutrition interventions only | 49 | 72 |
| Nutrition interventions in context of school improvement | 56 | 72 |
| Nutrition interventions in context of common school quality and labour market opportunities | 96 | 72 |

Productivity returns (Millions 2020 US$) and benefit cost ratios of scaling 10 early nutrition interventions, with and without changes to context

|  | **Quintile** | | | | | **Total** |
| --- | --- | --- | --- | --- | --- | --- |
|  | **1** | **2** | **3** | **4** | **5** |  |
| **Proportion of children per quintile** | 0.36 | 0.27 | 0.16 | 0.11 | 0.09 | **1** |
| **Present value** | | | | | | |
| Nutrition interventions only | 911.50 | 582.75 | 3610.48 | 206.82 | 164.01 | 5475.57 |
| Nutrition interventions in context of 50% school quality improvement | 1040.00 | 660.99 | 4082.40 | 227.60 | 164.01 | 6175.00 |
| Nutrition interventions in context of equal school quality and labour market participation | 1777.18 | 1123.02 | 6915.36 | 300.33 | 164.01 | 10279.90 |
| **Cost benefit ratios: Value of returns per US$1 invested in nutrition interventions** | | | | | | |
| Nutrition interventions only | 16 | 16 | 16 | 22 | 23 | 18 |
| Nutrition interventions in context of 50% school quality improvement | 18 | 18 | 18 | 24 | 23 | 20 |
| Nutrition interventions in context of common school quality and labour market participation | 31 | 31 | 30 | 32 | 23 | 30 |
