# Supplementary material for: Realising the potential human development returns to investing in early and maternal nutrition: The importance of identifying and addressing constraints over the life course
Source: PLOS Glob Public Health. 2021 Oct 13;1(10):e0000021. doi: 10.1371/journal.pgph.0000021 (PMC10022083; doi:10.1371/journal.pgph.0000021)
Supplement: S6 Appendix — (DOC) [file pgph.0000021.s006.doc]

## **S1 appendix 6: supplementary material references**

1. National Department of Health (NDoH), Statistics South Africa (Stats SA), South African Medical Research Council (SAMRC), and ICF[Producers]. South Africa Demographic and Health Survey 2016[dataset]. Pretoria, South Africa, and Rockville, Maryland, USA: NDoH, Stats SA, SAMRC, and ICF [Distributers]; 2019. <https://dhsprogram.com/data/dataset/South-Africa_Standard-DHS_2016.cfm?flag=0>
2. National Department of Health (NDoH), Statistics South Africa (Stats SA), South African Medical Research Council (SAMRC), and ICF. South Africa Demographic and Health Survey 2016. Pretoria, South Africa, and Rockville, Maryland, USA: NDoH, Stats SA, SAMRC, and ICF.; 2019. <https://dhsprogram.com/pubs/pdf/FR337/FR337.pdf> [no DOI]
3. UNAIDS. Country Factsheets South Africa. 2019. <https://www.unaids.org/en/regionscountries/countries/southafrica> (accessed 29 April 2020).
4. Statistics South Africa. Vulnerable Groups Series III Report: The social profile of children aged 7-17 years, 2002-2016. Pretoria: Statistics South Africa, 2019. [http://www.statssa.gov.za/publications/Report%2003-19-04/Report%2003-19-042016.pdf](http://www.statssa.gov.za/publications/Report 03-19-04/Report 03-19-042016.pdf)
5. Statistics South Africa. Earnings and spending in South Africa, 2006-2011. Pretoria: Statistics South Africa; 2015. <http://www.statssa.gov.za/publications/Report-01-11-02/Report-01-11-022006.pdf>
6. Statistics South Africa. South Africa Demographic and Health Survey 2016: Key Indicator Report, SR248. Pretoria: Statistics South Africa, 2019. [https://www.statssa.gov.za/publications/Report%2003-00-09/Report%2003-00-092016.pdf](https://www.statssa.gov.za/publications/Report 03-00-09/Report 03-00-092016.pdf)
7. Equity Technical Working Group of Countdown to 2030 and the team at the International Center for Equity in Health, Federal University of Pelotas. South Africa Profile. Countdown to 2030; 2019. [https://www.countdown2030.org/pdf/South-Africa-2016-DHS.pdf](https://www.countdown2030.org/pdf/South-Africa-2016-DHS.pdf (5)  (accessed 5 April 2020).
8. Statistics South Africa. Inequality Trends in South Africa: a multidimensional diagnostic of inequality. Pretoria: Statistics South Africa; 2019. <http://www.statssa.gov.za/publications/Report-03-10-19/Report-03-10-192017.pdf>
9. World Bank. World Bank Open Data. <https://data.worldbank.org/> (accessed 5 April 2020).
10. Department of Public Service and Administration South Africa. S1 appendix A to DPSA Circular 10 of 2018: Salary scales, with translation keys, for employees on salary levels 1 to 12 and covered by Occupation Specific Dispensations (OSD). Pretoria: Department of Public Service and Administration; 2018. [http://www.dpsa.gov.za/dpsa2g/r_documents.asp#models](http://www.dpsa.gov.za/dpsa2g/r_documents.asp#models)
11. Statistics South Africa. Consumer Price Index. Pretoria: STASSA; 2020. <http://www.statssa.gov.za/publications/P0141/P0141December2020.pdf>
12. Fink G, Peet E, Danaei G, et al. Schooling and wage income losses due to early-childhood growth faltering in developing countries: National, regional, and global estimates. Am J Clin Nutr. 2016; 104: 104-112. <https://pubmed.ncbi.nlm.nih.gov/27357091/>
13. Adair LS, Fall CH, Osmond C, et al. Associations of linear growth and relative weight gain during early life with adult health and human capital in countries of low and middle income: findings from five birth cohort studies. Lancet. 2013;382:525-534. doi: 10.1016/S0140-6736(13)60103-8. <https://pubmed.ncbi.nlm.nih.gov/23541370/>
14. Salisbury T. Education and inequality in South Africa: returns to schooling in the post-apartheid era. Int J Educ Dev. 2016;46:43-52. doi: 10.1016/j.ijedudev.2015.07.004. <https://www.sciencedirect.com/science/article/abs/pii/S0738059315000802>
15. Van Der Berg S, Burger C, Burger R, et al. Low quality education as a poverty trap Low quality education as a poverty trap. Stellenbosch Economic Working Papers: 25/11. Stellenbosch: Department of economics and the bureau for economic research at the University of Stellenbosch; 2011. <https://www.ekon.sun.ac.za/wpapers/2011/wp252011/wp-25-2011.pdf>
16. Department of Basic Education South Africa. Report on the 2019 National Senior Certificate Examination, let’s grow South Africa together. Pretoria: DoBE; 2020. https://www.education.gov.za/Portals/0/Documents/Reports/2019 NSC Examination Report.pdf?ver=2020-01-07-155811-230. [https://www.education.gov.za/Portals/0/Documents/Reports/2019%20NSC%20Examination%20Report.pdf?ver=2020-01-07-155811-230](https://www.education.gov.za/Portals/0/Documents/Reports/2019 NSC Examination Report.pdf?ver=2020-01-07-155811-230)
17. Statistics South Africa. General Household Survey, 2018- Statistical Release P0318. Pretoria: STATSSA; 2018. <http://www.statssa.gov.za/publications/P0318/P03182018.pdf>.
18. Alderman H, Behrman JR, Puett C. Big numbers about small children: estimating the economic benefits of addressing undernutrition. World Bank Res Obs. 2017;32:107-125. doi: 10.1093/wbro/lkw003. <https://academic.oup.com/wbro/article/32/1/107/2670361>
